# Alteration of nephrocystins and IFT-A proteins causes similar ciliary phenotypes leading to Nephronophthisis

**DOI:** 10.1186/2046-2530-1-S1-P99

**Published:** 2012-11-16

**Authors:** S Saunier, AA Bizet, F Silbermann, E Filhol, T Blisnick, A Henneveu, E Montenont, I Perrault, C Boyle-Feysot, J-M Rozet, P Bastin, HH Arts, C Antignac, AR Benmerah

**Affiliations:** 1 Inserm U983, France; 2 Pasteur Institute, CNRS UMR2581, France; 3 Inserm U1016, Cochin Institute, France; 4 Inserm U781, France; 5 Fondation Imagine, Paris, France; 6 Radboud University Nijmegen Medical Center, the Netherlands

## 

Nephronophtisis (NPH) is a kidney ciliopathy often associated with extra-renal defects and for which 12 genes (*NPHP1-12*) have been identified. NPHP1 and NPHP4 control the ciliary access at the transition zone and the velocity of some intraflagellar transport (IFT)/BBS proteins in *C.elegans*. Recently, in a collaborative effort, we have identified, in families with isolated NPH, mutations in *TTC21B* as well as in *WDR19*, which encode the retrograde IFT-A proteins IFT139 and IFT144, respectively. By ciliome sequencing of 1600 candidate genes from 14 NPH patients followed by Sanger sequencing of a cohort of 52 patients, we have found respectively 8 and 7 patients carrying pathogenic missense mutations in genes coding IFT-A proteins, including *WDR35*, *TTC21B* and *IFT140*, which could partially affect their function. Together, these results indicate that IFT-A are involved in nephronophtisis. Moreover, alteration of cilia length was observed in patient kidney, *Nphp4-/-* mice kidney tubules and *NPHP1* or *NPHP4* knockdown IMCD3 cell lines. In these cells, primary cilia present swellings at the distal region accompanied by an accumulation of IFT-B at the base and the tip, similar to what was observed in IFT-A mutants, suggesting a possible alteration of retrograde transport. Additionally, ARL13B, a small GTPase required for proper cilium shape and IFT stability, is absent along the axoneme of NPHP4-KD-IMCD cells. By controlling the entry of ciliary components at the transition zone, NPHP1 and NPHP4 may modulate IFT-A cargos thus participating in the same pathway (i.e. Wnt/PCP), alteration of which would lead to renal lesions observed in nephronophthisis.

